# The chicken or the egg? Exploring bi-directional associations between Newcastle disease vaccination and village chicken flock size in rural Tanzania

**DOI:** 10.1371/journal.pone.0188230

**Published:** 2017-11-16

**Authors:** Julia de Bruyn, Peter C. Thomson, Brigitte Bagnol, Wende Maulaga, Elpidius Rukambile, Robyn G. Alders

**Affiliations:** 1 School of Life and Environmental Sciences, University of Sydney, Sydney, NSW, Australia; 2 Charles Perkins Centre, University of Sydney, Sydney, NSW, Australia; 3 International Rural Poultry Centre, KYEEMA Foundation, Brisbane, QLD, Australia; 4 Department of Anthropology, University of Witswatersrand, Johannesburg, South Africa; 5 Central Veterinary Laboratory, Tanzania Veterinary Laboratory Agency, Dar es Salaam, Tanzania; University of California Davis, UNITED STATES

## Abstract

Newcastle disease (ND) is a viral disease of poultry with global importance, responsible for the loss of a potential source of household nutrition and economic livelihood in many low-income food-deficit countries. Periodic outbreaks of this endemic disease result in high mortality amongst free-ranging chicken flocks and may serve as a disincentive for rural households to invest time or resources in poultry-keeping. Sustainable ND control can be achieved through vaccination using a thermotolerant vaccine administered via eyedrop by trained “community vaccinators”. This article evaluates the uptake and outcomes of fee-for-service ND vaccination programs in eight rural villages in the semi-arid central zone of Tanzania. It represents part of an interdisciplinary program seeking to address chronic undernutrition in children through improvements to existing poultry and crop systems. Newcastle disease vaccination uptake was found to vary substantially across communities and seasons, with a significantly higher level of vaccination amongst households participating in a longitudinal study of children’s growth compared with non-participating households (*p* = 0.009). Two multivariable model analyses were used to explore associations between vaccination and chicken numbers, allowing for clustered data and socioeconomic and cultural variation amongst the population. Results demonstrated that both (a) households that undertook ND vaccination had a significantly larger chicken flock size in the period between that vaccination campaign and the next compared with those that did not vaccinate (*p* = 0.018); and (b) households with larger chicken flocks at the time of vaccination were significantly more likely to participate in vaccination programs (*p* < 0.001). Additionally, households vaccinating in all three vaccination campaigns held over 12 months were identified to have significantly larger chicken flocks at the end of this period (*p* < 0.001). Opportunities to understand causality and complexity through quantitative analyses are limited, and there is a role for qualitative approaches to explore decisions made by poultry-keeping households and the motivations, challenges and priorities of community vaccinators. Evidence of a bi-directional relationship, however, whereby vaccination leads to greater chicken numbers, and larger flocks are more likely to be vaccinated, offers useful insights into the efficacy of fee-for-service animal health programs. This article concludes that attention should be focused on ways of supporting the participation of vulnerable households in ND vaccination campaigns, and encouraging regular vaccination throughout the year, as a pathway to strengthen food security, promote resilience and contribute to improved human nutrition.

## Introduction

Small flocks of poultry are kept by rural and periurban households throughout low-and middle-income countries, where their contributions span income generation, resilience in times of financial need, provision of animal-source foods, female empowerment and sociocultural activities [1–5]. These genetically-diverse chickens are well-suited to low-input production systems: scavenging for feed, hatching eggs, raising chicks, and sometimes roosting in trees overnight. Constraints to village poultry production include high mortality rates due to disease and predation, and a reliance on available environmental food resources [6].

Newcastle disease (ND) is a viral disease of poultry with global importance, responsible for the loss of economic livelihood and a potential source of household nutrition in many resource-poor settings [7]. Periodic outbreaks result in high mortality amongst free-ranging flocks and serve as a disincentive for poultry-keepers to invest time or resources in their birds. Since opportunities for biosecurity approaches are limited in village settings, where chickens commonly move through the village environment, pass through markets and are given to visiting guests, ND control in village poultry systems is heavily reliant on vaccination.

A successful ND vaccination program has been proposed to require a minimum of 85% of a flock to receive an adequate dose and respond appropriately to the vaccine, in order to achieve herd immunity [8]. This is yet to be validated in village settings, where suboptimal conditions may include nutritional deficiencies, stress, immunosuppression and repeated viral challenges [9]. Amongst the spectrum of ND vaccines currently available and under evaluation, live vaccines developed from low-virulence viral strains remain the mainstay of ND control in many rural communities of Africa. Of these, the I-2 ND vaccine has advantages of increased thermotolerance (an important characteristic in the absence of a reliable cold chain), being safe to administer to birds of all ages including day old chicks, and safe when administered in excess of the recommended dose. The I-2 ND vaccine master seed has been made freely available to low- and middle-income countries to allow a vaccine suitable for use village poultry flocks to be produced locally [10].

Efforts to achieve sustainable ND control in resource-poor settings have included the development of a “community vaccinator” model, whereby a participatory training program and culturally-appropriate extension materials are used to equip local people to offer fee-for-service vaccination through coordinated four-monthly campaigns [11]. Community-based approaches have been central to the delivery of human health care [12, 13] and conservation programs [14–16] for many years, however their use in animal health is less common. Although village poultry systems pose specific challenges to disease control, the community structure of rural African villages is recognised as being conducive to collective action–such as programs to improve chicken health and production–in a way which is less possible in high-income, urban settings [17].

This article presents findings following the implementation of community-based ND vaccination programs using the I-2 ND vaccine in rural communities in Tanzania [18]. It represents part of a nutrition-sensitive program, exploring opportunities to strengthen household nutrition through improvements to existing poultry and crop systems, with a focus on assets controlled and managed by women. In this paper, we describe levels of chicken ownership and chicken flock size over a two-year period and explore levels of vaccination uptake (across all households, and within a randomly selected subset participating in a longitudinal study assessing children’s height-for-age). We test associations between the availability of vaccination at a village level and chicken flock sizes, and test bi-directional associations between chicken numbers and vaccination at a household level.

## Methods

### Ethics approval

The study design, protocols and research instruments for this program were approved by the National Institute for Medical Research ethics committee (NIMR/HQ/R.8a/Vol.IX/1690) in Tanzania, The University of Sydney Human Research Ethics Committee (2014/209) and The University of Sydney Animal Ethics Committee (2013/6065). All participants provided informed consent prior to participating in the study, with assurance of confidentiality, anonymity, voluntary participation and no adverse effects in case of refusal.

### Study area and population

This longitudinal study comprises eight rural villages in two adjoining wards of Manyoni District in the semi-arid central zone of Tanzania, and represents a subset of a cluster randomised controlled trial evaluating the impact of village poultry vaccination programs and strategic improvements to crop systems on levels of chronic undernutrition in children [18]. Study sites were selected based on recommendations of in-country partners, according to levels of child stunting and the absence of existing human nutrition or poultry vaccination programs. Within the district, a unimodal pattern of rainfall is expected between November and April, with long-term data indicating mean annual rainfall of 624 mm (SD = 179 mm) and a mean number of rain days of 49 (SD = 15) [19].

A ward census was conducted by the project in April 2014 in Sanza Ward and October 2014 in Majiri Ward, with enumerators registering details of all household members, ownership of chickens and interest in chicken-keeping. Eligibility criteria for inclusion in the longitudinal study included the presence of a child under 24 months of age, and either current ownership of chickens or an intention to keep chickens within a two-year period. Few households were excluded based on the latter criterion.

**Fig 1:**
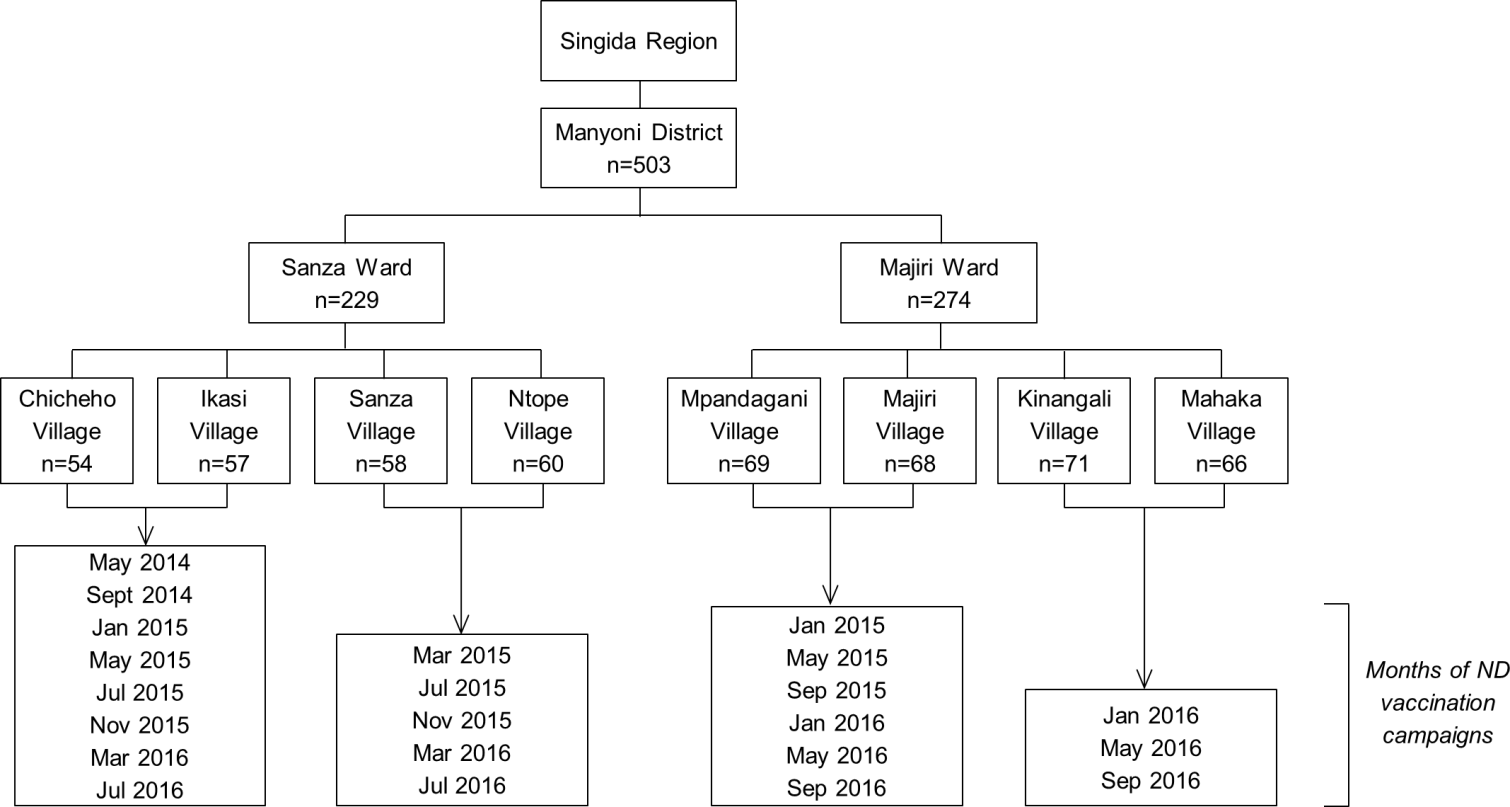
Overview of administrative units in study population, with the number of enrolled households in each of eight villages, and aggregated at ward and district levels. The timing of vaccination campaigns is shown.

Two-stage sampling was used to give a total of 240 households in Sanza Ward and 280 in Majiri Ward: by first enrolling all eligible households with a child under 12 months of age, and then using random selection to enrol additional households with a child aged 12–24 months. Baseline data collection was completed for 229 households in Sanza Ward in May 2014 and 274 households in Majiri Ward in November 2014, as part of the staged implementation within the larger project design. An overview of administrative units within the study and the number of enrolled households at a village, ward and district level are shown in Fig 1.

### Study intervention

Newcastle disease (ND) control programs using the thermotolerant I-2 ND vaccine, administered via eyedrop [20] were established within the project sites, beginning in May 2014 in the first two of the eight villages (Fig 1). Local candidates for the role of “community vaccinator” were identified in consultation with village leaders. Training workshops were conducted using materials developed for and pre-tested in resource-poor settings [21]. These included both theoretical and practical components to cover aspects of chicken health and disease, principles of ND transmission and control, vaccine storage and handling, and logistical aspects of implementing community-wide vaccination campaigns.

In addition to the six-month delay between commencement of research activities between the two wards, there was a staggered start to ND vaccination programs within wards. At ward-level meetings in both Sanza and Majiri, village representatives drew pieces of paper at random to designate their village for immediate or delayed introduction of ND vaccination, with a delay of three campaigns between the two groups (Fig 1). Prior to each campaign, community vaccinators visited households to register chicken numbers and establish chicken-keepers’ interest in fee-for-service vaccination, allowing an appropriate quantity of vaccine to be ordered. The cost of vaccination was initially set at 50 Tanzanian Shillings (TZS) per bird in all villages, and was increased to 100 TZS per bird in Majiri Ward in 2016 based on local consensus.

The months of vaccination campaigns within the eight villages is shown in Fig 1. Studies on the epidemiology of ND in village chickens are limited, however general information from Veterinary Investigation Centres across different agroecological zones of Tanzania [22] suggests a heightened risk of ND outbreaks between July and November each year, during the dry season. Accordingly, initial recommendations were that vaccination campaigns be held in January, May and September. This timing was followed in Majiri Ward throughout the study. In Sanza Ward, vaccination months were changed to March, July and November in the second year of implementation. This transition was made both to reflect the perceived risk of outbreaks within the community, and to accommodate changes to the Tanzanian financial year in Tanzania and associated logistical challenges of distributing vaccine in January.

Although it is considered prudent to conduct two ND vaccination campaigns prior to the high-risk period for disease outbreaks [23], delays in project inception led to the first campaign (in Chicheho and Ikasi villages) being held in late May 2014. This coincided with reports of illness and mortality in a small number of chickens within the area, suggesting the potential presence of a disease compatible with ND. A decision was made to proceed with the campaign, given it was considered a minor risk that chicken-keepers might attribute post-vaccination illness or mortality in their chickens either to the inefficacy of the vaccine or as a direct outcome of vaccination. In addition to hand-washing between each household visited, payment was postponed to a follow-up visit as an additional measure to reduce the potential for disease transmission, with the transfer of money identified as a potential pathway for viral transmission.

### Data sources

Information used for this analysis falls into two broad categories: (1) relating to all households to which ND vaccination was made available, and (2) relating to the subset of households enrolled in the longitudinal study of children’s growth (hereafter referred to as “enrolled households”). In the former category, the total number of households was derived from initial census data collected by the project, and the number of households vaccinating their chickens in each campaign was determined from community vaccinators’ records. Daily rainfall data were recorded from a rain gauge with 1 mm graduations, located at the village office in Ikasi Village, Sanza Ward and Kinangali Village, Majiri Ward.

Information on study participants was drawn from two questionnaires, collected through interviews by local enumerators recruited and trained within each ward. One questionnaire (applied at six-monthly intervals to mothers of enrolled children), focussed on maternal and child health and nutrition, while the other (applied annually to an intended equal number of male and female household members; actual sample comprised 60.5% female respondents of 1,354 completed questionnaires) encompassed demographic data, socioeconomic factors, livelihoods and chicken-keeping practices. Additionally, two representatives from each village (one male, one female) were employed as “Community Assistants” to collect ongoing data from enrolled households. The number of chickens owned, categorised by age (i.e. under or over two months) and participation in each vaccination campaign were recorded during twice-monthly household visits.

### Data analysis

#### Defining variables

Socioeconomic status of enrolled households was determined using a modified version of a “household domestic assets index” (HDAI), developed for use in sub-Saharan Africa [24]. The index assigns a weight to livestock and non-livestock assets according to their equivalent value. It is acknowledged that the relative value of assets will vary between settings, however the reference’s weighting system was considered adequately appropriate for the study sites to provide a reasonable estimate of enrolled households’ wealth. Given that some of the information involved in index construction (such as the size of land ownership and the age of assets) was not collected within this study, a modified index was formed based on livestock species and household items owned. Chicken numbers were excluded from these calculations, in order to evaluate their influence on vaccination uptake separately to their general contribution to household wealth.

Language group, as a proxy for ethnicity and cultural practices, was also considered as a potential determinant of chicken ownership and vaccination uptake, based on observed and documented differences in household dynamics, diets and the practice of agropastoralism in this setting [25, 26]. Questionnaire responses for the “first language” of both the mother and father of children participating in the study were combined with information on the gender of the household head to determine the dominant language group of each household, likely to influence agricultural and dietary practices.

The number of chickens owned by households was recorded at two-weekly intervals. While chickens of all ages were included in descriptive summaries, the number of chickens over two months of age was used as a predictor and outcome variable in inferential analyses. This distinction was made based on documented high rates of mortality amongst chicks in village settings from causes other than ND, such as predation, harsh weather conditions and poor nutrition [27, 28].

#### Descriptive statistics

Percentages were determined for categorical variables and means and standard deviations or medians and interquartile ranges (IQR) calculated, for normally and non-normally distributed continuous variables, respectively. Records of chicken numbers in enrolled households were aggregated at village and ward levels for twice-monthly intervals over a two year period in both wards (beginning in July 2014 in Sanza Ward, and November 2014 in Majiri Ward). Graphical summaries were assembled for the percentage of enrolled households owning chickens and the average chicken flock size over time. In recognition of fluctuating levels of village chicken ownership, the percentage of households owning chickens consistently over a twelve-month period, and those owning chickens intermittently or not at all, were evaluated for each ward.

Levels of vaccination uptake were determined for all households in a village, and amongst households enrolled in the study. In the former case, given the absence of information on chicken ownership across the whole village, vaccination levels were calculated relative to the total number of households recorded in the census (a proportion of which it is noted would not be keeping chickens). For the enrolled subset, households owning chickens were categorised as vaccinating or non-vaccinating according to their participation in each campaign.

#### Univariable and multivariable models

Depending on the type of response variable, linear mixed models (for quantitative variables) or generalised linear mixed models (for binary and count variables) were used, with the mixed model approach to allow for geographical clustering. For all analyses, univariable models were first used to test unconditional associations between predictor variables of interest (including socioeconomic and demographic characteristics and temporal factors) and the two outcomes of interest: chicken flock size and vaccination uptake. In the case of chicken numbers and asset scores, log-transformations were used to minimise the excessive influence of very large numbers. Variables with *p*-values under 0.1 were included in multivariable models, and a manual backwards elimination approach used with variables being retained if they were significant at the 5% level. All multivariable models included ward, village, subvillage and household identification as random effects, to allow for clustered data.

**Fig 2:**
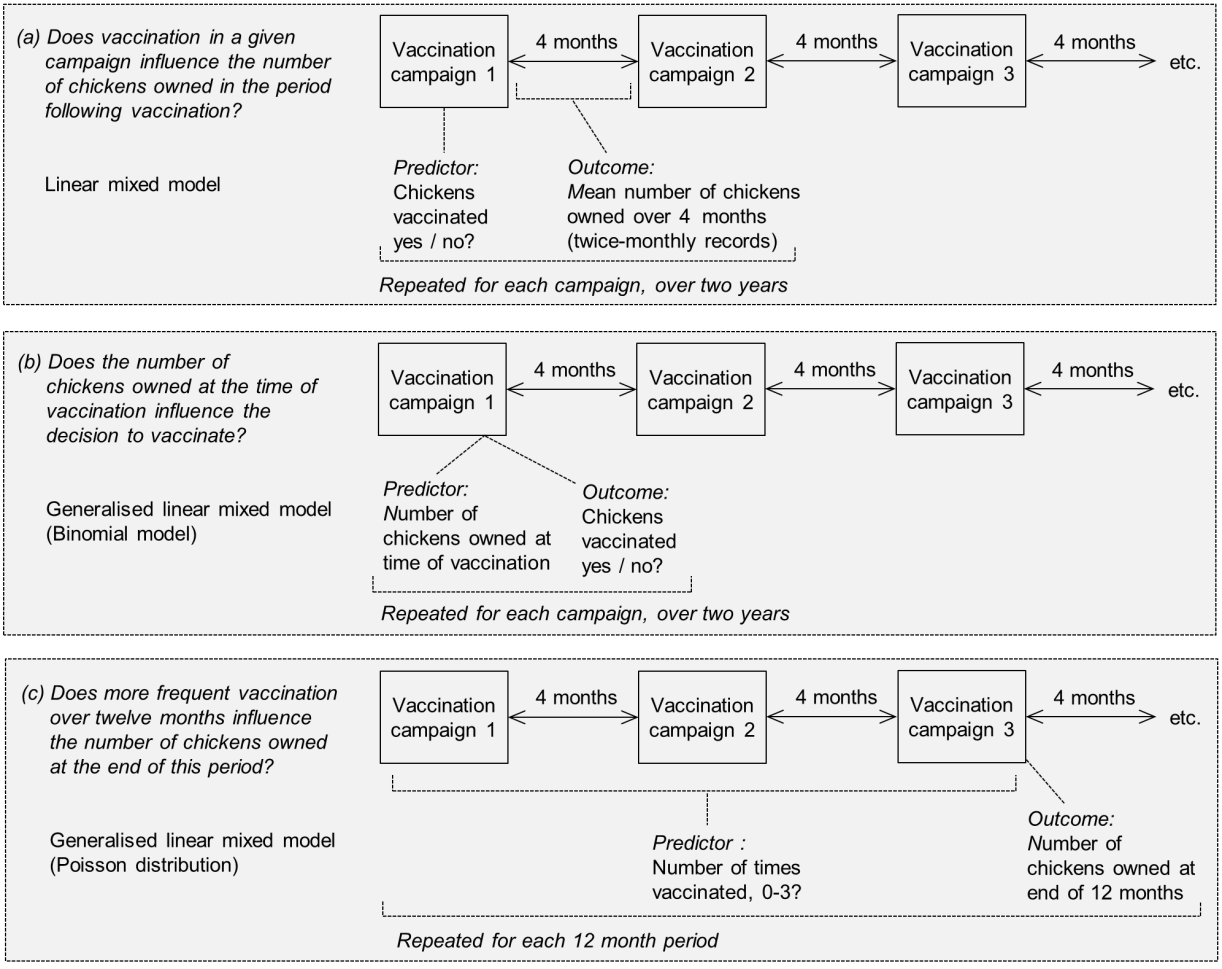
Overview of analyses exploring associations between chicken vaccination and chicken numbers, with time periods for relevant predictor and outcome variables. All were within multivariable models.

Initial analyses explored: (a) longitudinal associations between enrolment in the study and participation in ND vaccination campaigns, and (b), for enrolled households, associations between the timing of commencement of vaccination (immediate or delayed) and levels of participation in the first campaign. The relationship between chicken numbers and ND vaccination was recognised as having the potential to be bi-directional: (1) with lower mortality amongst vaccinated birds resulting in increased chicken flock size, and / or (2) with households owning more chickens being more likely to invest in vaccination. Schematic diagrams of analyses conducted, including the relevant time frames to explore causality, are shown in Fig 2.

Vaccination in a given campaign was evaluated as a predictor of chicken flock size in the period following vaccination, by considering the mean number of chickens owned between one campaign and the next (Fig 2A; linear mixed model). To explore the alternative causal pathway, associations between chicken flock size and the decision to vaccinate were tested using the number of chickens owned at the time of vaccination (Fig 2B; generalised linear mixed model (binomial model)). Finally, the significance of participation in multiple vaccination campaigns over twelve months was tested as a predictor of the chicken flock size at the end of this period (Fig 2C; generalised linear mixed model (Poisson distribution)). The fit of the mixed models was assessed using standard residual diagnostic plot methodologies. All analyses were conducted using GenStat Release 18 (https://www.vsni.co.uk/).

## Results

### Characterising the study population

Demographic and socioeconomic characteristics of enrolled households were compiled using baseline data from each of the two wards (Table 1). The percentage of households identifying as having a female household head varied significantly between wards (*p* < 0.001), from 16.4% in Majiri to 30.2% in Sanza. Household size ranged from two to 21 people across the study sample, with a median of five household members in both wards. Fourteen language groups were represented, based on questionnaire responses for the “first language” of both the mother and father of enrolled children. Gogo constituted the most common language group (*n* = 381), with Sukuma the second most common (*n* = 46).

**Table 1:** Overview of household characteristics, by ward.

	Sanza Ward	Majiri Ward
Baseline data collection	May 2014	November 2014
Enrolled households (*n*)	229	274
Female-headed households (%)	30.2^a^	16.4^a^
Household size		
Mean (SD)	5.4 (1.9)	5.5 (2.6)
Range	2–11	2–21
Language group (%)		
Gogo	78.2^a^	74.8^a^
Sukuma	6.1^a^	14.6^a^
Other	4.4	2.6
Unspecified	11.4^a^	8.0^a^
Household Domestic Assets Index		
Median (IQR)	12 (5–50)^b^	26 (7–115)^b^
0–49 (%)	74.7	59.6
50–99 (%)	10.7	13.1
≥100 (%)	14.7	27.3
Livestock ownership at baseline (%)		
Cattle	26.7^a^	36.2^a^
Sheep or goats	27.1^a^	47.8^a^
Chickens	51.1^a^	42.1^a^
Number of livestock^c^, median (IQR)		
Cattle	4 (2–17)^b^	10 (4–20)^b^
Sheep or goats	14 (7–20)	12 (5–25)
Chickens	7 (2–13)	8 (5–13)

Significant differences between wards (*p* < 0.05), as determined by

^a^ Chi-square tests and

^b^ t-tests.

^c^ Amongst households keeping livestock, for each category

The modified HDAI, based on the sum of weighted livestock and non-livestock assets, suggested a wide variation in socioeconomic status amongst study participants. The range of HDAI scores extended from zero to 1,010, with a prominently positively-skewed distribution. Significant variation was seen between wards (*p* = 0.002), from a median HDAI of 12 in Sanza and 26 in Majiri. The percentage of households owning ruminants also varied significantly between wards (*p* = 0.024 for cattle, *p* < 0.001 for sheep and goats), and cattle herds were significantly larger in Majiri Ward (*p* = 0.012). Chickens were identified to be owned by 51.1% of enrolled households in Sanza Ward and by 42.1% in Majiri Ward at the time of baseline data collection. The known seasonality of village chicken numbers and differing months in which baseline assessments were conducted prevents conclusions being drawn about significance of differing levels of chicken ownership using this data.

### Chicken ownership and flock size in enrolled households

**Fig 3:**
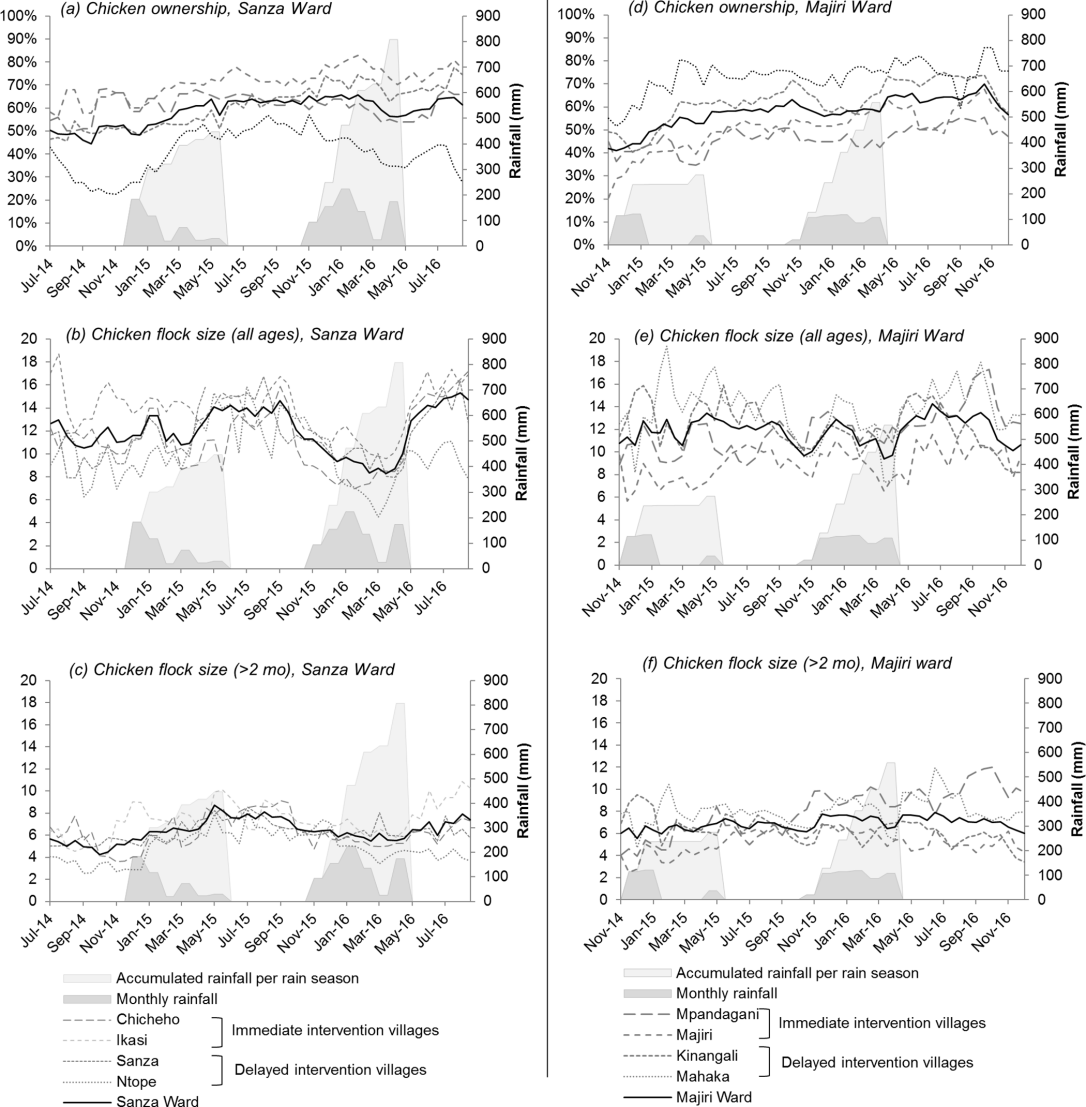
Percentage of enrolled households owning chickens (a, d) and mean flock size per household, including chickens of all ages (b, e) and restricted to chickens over two months of age (c, f), with monthly and accumulated rainfall per year.

Twice-monthly records of chicken numbers in enrolled households were aggregated at the village and ward level, to evaluate trends in chicken ownership and chicken flock size over a two-year period beginning in July 2014 in Sanza Ward and November 2014 in Majiri Ward (Fig 3). Flock size was evaluated both in terms of the total number of chickens (Fig 3B and 3E), and the number older than two months of age (i.e. excluding chicks; Fig 3C and 3F). Daily rainfall data are shown as monthly and accumulated totals for each rain season, to reflect seasonality within the agricultural calendar in the study sites.

There was a mild increase in the percentage of enrolled households keeping chickens in Sanza Ward (from 50.2% to 59.3% over 24 months; Fig 3A), with considerable variation between villages. Levels of chicken ownership increased in two villages (from 58.3% to 71.4% in Ikasi, and 46.4% to 68.6% in Sanza), but remained relatively unchanged in the other two. The most marked variation during the data collection period was seen in Ntope Village, one of the delayed vaccination villages, where chicken ownership levels dropped to 22.6% in November 2014, rising to 56.9% by November 2015, before falling to 34.7% in April 2016.

In Majiri Ward, an upward trend in levels of chicken ownership amongst enrolled households was evident (from 42.0% to 66.2% over 24 months; Fig 3D). At a village level, fewer households kept chickens in the immediate vaccination communities, compared to the delayed vaccination communities, both at the time of commencement of project activities and throughout the period of data collection. Levels of chicken ownership were highest in Mahaka Village, while Majiri Village recorded the lowest levels, but the greatest increase, over 24 months (from 20.6% to 65.0%). Fluctuations in levels of chicken ownership within villages of Majiri Ward were less prominent than in Sanza Ward, during the respective data collection periods.

In November 2014, the first point at which information is available for both study sites, comparable mean flock sizes were documented in the two wards (11.0 in Sanza vs 10.8 in Majiri, total chicken numbers). Although limited to a 24 month period, data from Sanza Ward suggests the possibility of a seasonal pattern in chicken numbers, with a decrease in the mean flock size through the dry season and an increase through the wet season. A particularly marked decline in overall chicken numbers was seen between September 2015 and March 2016 (from a mean flock size of 14.6 to 8.3). Ikasi village, in which the percentage of chicken-owning households was highest, also recorded the greatest mean chicken flock size in Sanza Ward.

There was no suggestion of a seasonal or temporal effect on chicken numbers in Majiri Ward, where ward-level data showed a moderately stable flock size over the data collection period (range of 6.0–7.9 chickens above two months of age, over 24 months). As in Sanza Ward, the village with the highest level of chicken ownership in the ward, Mahaka, also had the largest mean number of chickens per household. Although only a relatively mild increase in the percentage of chicken-owning households was seen in Mpandagani village (from 44.9% to 53.7% of enrolled households, over 24 months), a marked increase in chicken flock size was recorded (from 4.0 to 12.0 chickens above two months of age).

In both study sites, more substantial fluctuations in mean flock size were evident when chicks (i.e. chickens under two months of age) were included in calculations (Fig 3B and 3E, cf. Fig 3C and 3F). The inclusion of chicks in graphical summaries serves to illustrate the variability in flock size which accompanies the hatching of clutches of eggs and subsequent loss of some chicks to the hazards of predation, disease or poor nutrition in a village setting.

**Fig 4:**
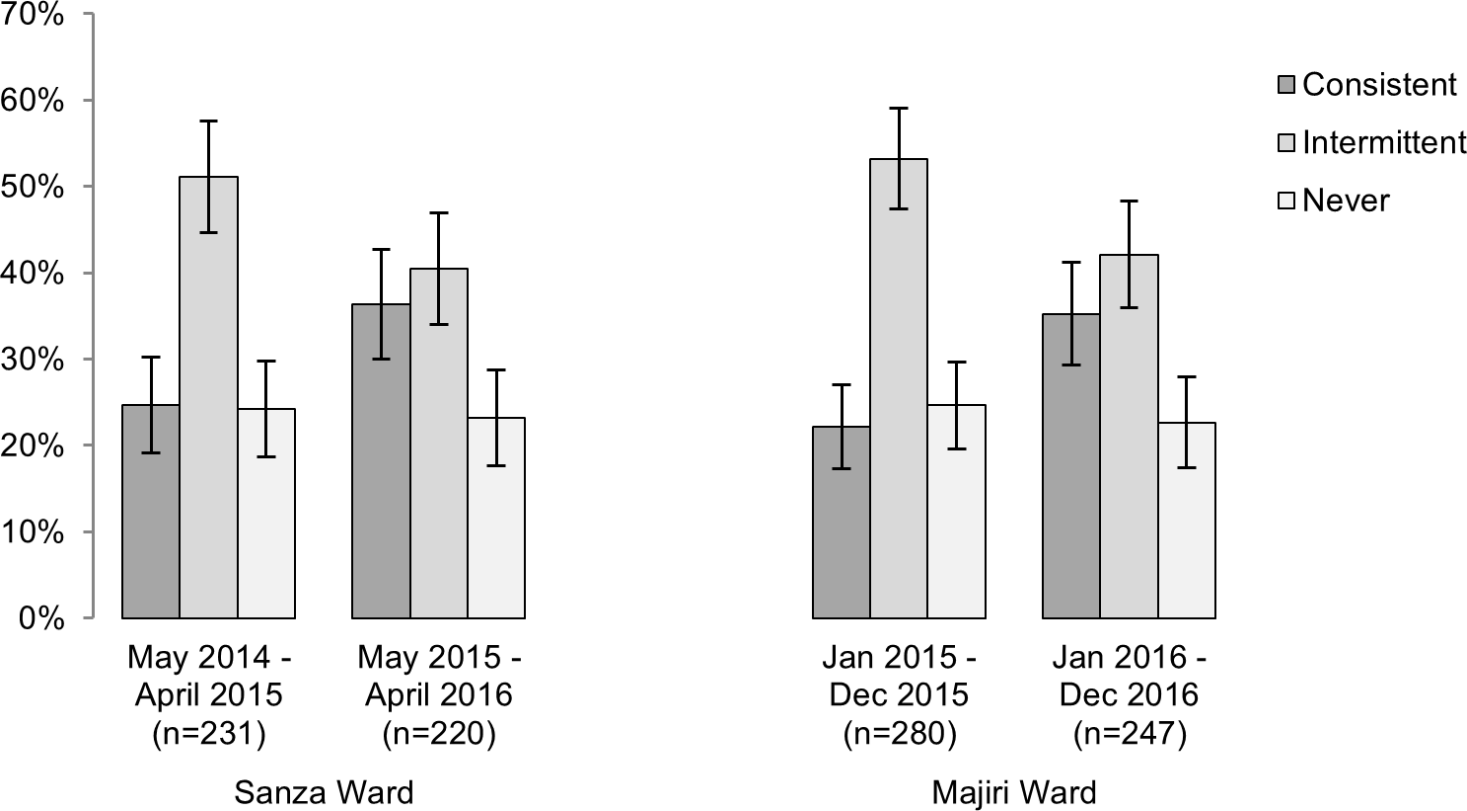
Percentage of enrolled households keeping chickens consistently, intermittently or not at all, during two consecutive twelve-month periods in Sanza and Majiri Ward. 95% confidence intervals are shown.

For all enrolled households with twice-monthly records available, the regularity with which chickens were kept was also considered. The percentage of households owning chickens consistently, intermittently and not at all, over consecutive twelve-month periods in each ward is presented in Fig 4. The percentage not keeping chickens has remained relatively stable over the two years evaluated (24.2% to 23.2% in Sanza Ward, 24.6% to 22.7% in Majiri Ward), however a moderate increase has been seen in the percentage of households consistently keeping chickens between one year and the next (24.7% to 36.4% in Sanza Ward, 22.1% to 35.2% in Majiri Ward). Of the relatively stable proportion of enrolled households owning chickens, it became more common to keep chickens consistently throughout the year during the second twelve-month period.

### Newcastle disease vaccination uptake

**Fig 5:**
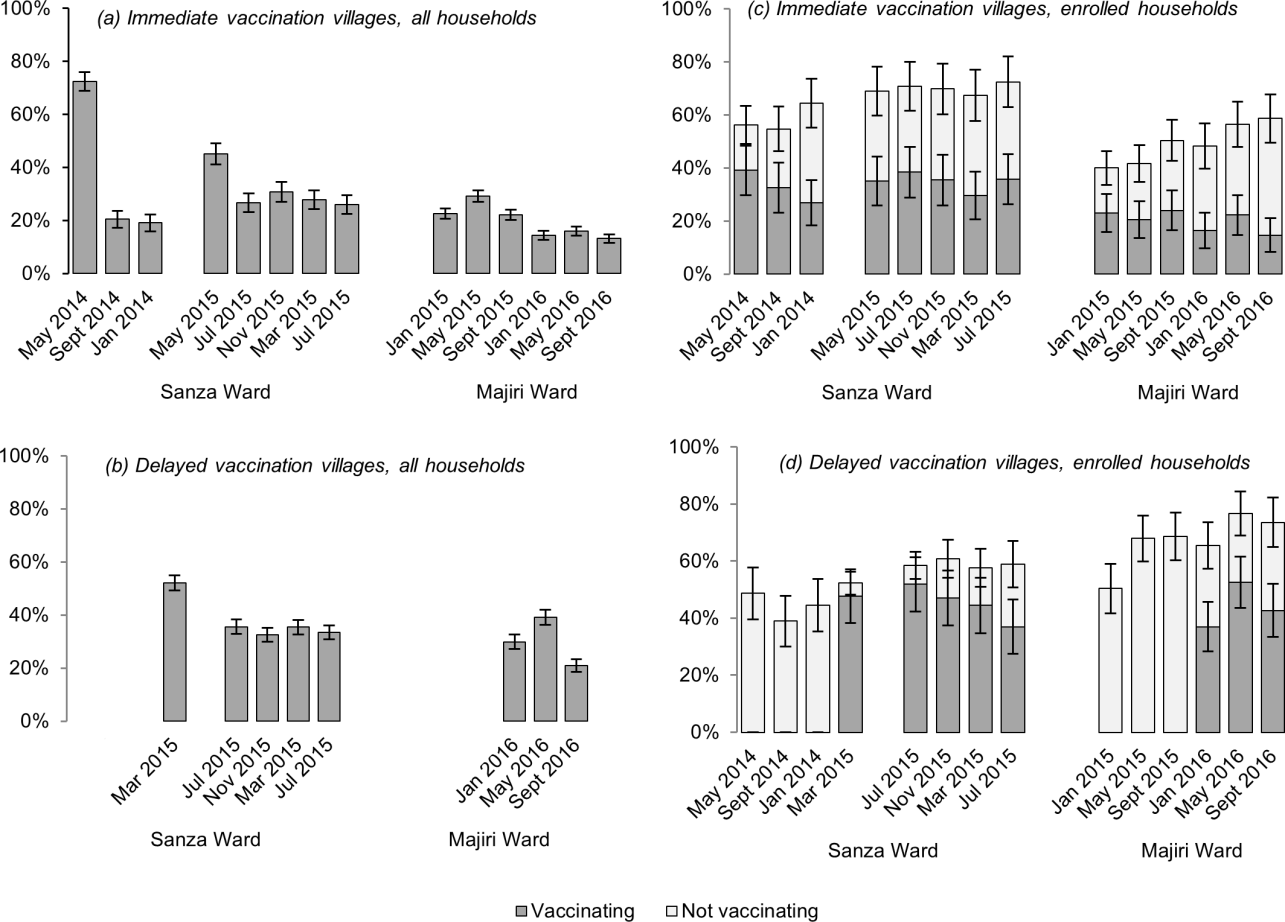
Percentage of households participating in each campaign (dark grey shading), according to intervention group: across all households (a and b), and amongst enrolled households (c and d). For enrolled households, the percentage owning chickens but not vaccinating is included for each campaign (light grey shading). 95% confidence intervals are shown.

Levels of uptake of ND vaccination varied substantially across wards, villages and campaigns. The percentage of households participating in each campaign has been evaluated across all households (Fig 5A and 5B), and amongst the subset of households enrolled in the longitudinal study (Fig 5C and 5D). Levels of chicken ownership across all households are not known, but for enrolled households, the percentage of households owning but not vaccinating chickens is also shown.

Vaccinator records indicate the first campaign in Sanza Ward, in May 2014, to have involved almost three-quarters of all households (72.4%) across the two intervention villages, but this reduced dramatically to around one-fifth of all households in the two subsequent campaigns (20.4% and 19.1% for September 2014 and January 2015, respectively). In the delayed intervention villages in Sanza Ward, around half of all households (52.1%) participated in the first campaign, in March 2015. At a ward level, vaccination levels have stabilised at just under one-third of all households for the four most recent campaigns evaluated (ranging from 30.9% in July 2016 to 32.9% in March 2016), with some fluctuation at a village level between one campaign and the next.

In Majiri Ward, vaccination uptake has been lower on average, compared to Sanza Ward (22.5% of all households versus 37.5%, across a two-year period in each ward), and with less marked variation at a ward level between campaigns (20.3–29.2%). Within both of the two years evaluated in Majiri Ward, the highest level of participation was recorded in the May campaign. Uptake of vaccination varied substantially between villages, with almost three times more households vaccinating in Kinangali compared to Mpandagani over the three campaigns in 2016 (30.8% versus 10.7%).

In the first campaign of the study, implemented in Chicheho and Ikasi villages in Sanza Ward in May 2014, comparisons between vaccination levels amongst enrolled households and across the broader community reveal a contrasting situation in the two intervention villages. In Ikasi village, similar uptake was seen in the two groups (52.1% vs. 50.9%) while in Chicheho, available data indicates 23.2% of enrolled households to have vaccinated chickens, compared to 87.7% of the wider community. This apparent under-representation of enrolled households has not been repeated in subsequent campaigns or alternative locations. In Majiri Ward, the most prominent difference between vaccination uptake was seen in Kinangali village in September 2016 (53.8% enrolled households vs. 26.1% in overall village).

To test the association between enrolment in the longitudinal study and vaccination of chickens, the number of enrolled households (vaccinating and total) was subtracted from community-wide data to give the number of “non-enrolled households” (vaccinating and total). In the May 2014 campaign in Chicheho village, records indicate the number of non-enrolled households vaccinating chickens to have *exceeded* the total number of non-enrolled households in the village. Excluding this campaign (for reasons elaborated in the Discussion), enrolment in the longitudinal study was identified as a significant predictor of chicken vaccination (*p* = 0.009). Controlling for variation between campaigns and clustering at the ward and village level, model-based predictions for vaccination participation were 36.2% (SE 3.9%) for enrolled households and 30.1% (SE 3.0%) for non-enrolled households.

The effect of the delay to the introduction of vaccination programs in some villages on participation in the first campaign was also explored using household-level data. Amongst enrolled households owning chickens during the month of vaccination, generalised linear mixed model analysis which included ward, village and subvillage locations as random effects indicated no significant difference in the probability of vaccinating in the first campaign where the vaccine was available immediately or those with a twelve month delay (*p* = 0.55).

### Linking Newcastle disease vaccination and chicken numbers

Relationships between ND vaccination and chicken numbers at a household level were explored using two years of data from enrolled households in villages where vaccination was available. The potential for differences between male- and female-headed households was considered in all models, but no significant associations were identified.

**Fig 6:**
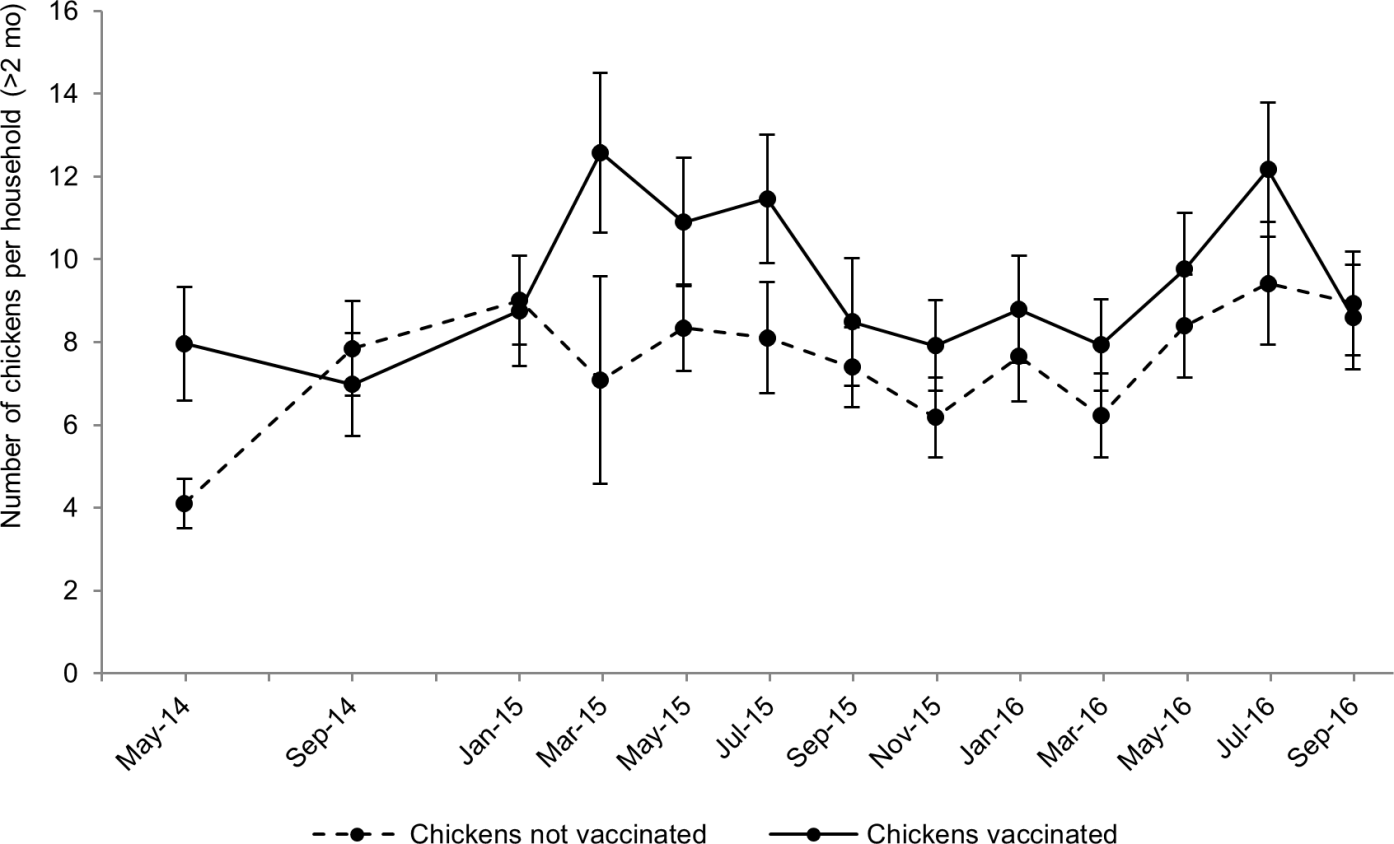
Model-based predictions for mean number of chickens (above two months of age) per household in the period between one vaccination campaign and the next, according to households’ participation in a given campaign (*p* = 0.025), based on multivariable analysis of enrolled households (controlling for language group and asset score).

Firstly, a linear mixed model was used to test associations between vaccination in a given campaign and the mean number of chickens owned in the period following vaccination (in most cases, the four month period between campaigns). Controlling for the influence of language group (with a predicted mean flock size of 10.8 (SE 1.6) chickens for Sukuma households, compared to 6.4 (SE 0.6) chickens for other language groups; *p* < 0.001) and HDAI score (*p* = 0.007), ND vaccination in a given campaign was significantly associated with greater chicken numbers in the period following vaccination (*p* < 0.001). The introduction of an interaction term to the model demonstrated that the association between vaccination and chicken flock size varied between campaigns (*p* = 0.025; Fig 6). Statistically significant differences between vaccinated and non-vaccinated flock sizes were demonstrated for the campaigns held in May 2014, March 2015 and July 2015. It is considered likely that limited statistical power prevented this same effect being demonstrated in other vaccination campaigns.

**Fig 7:**
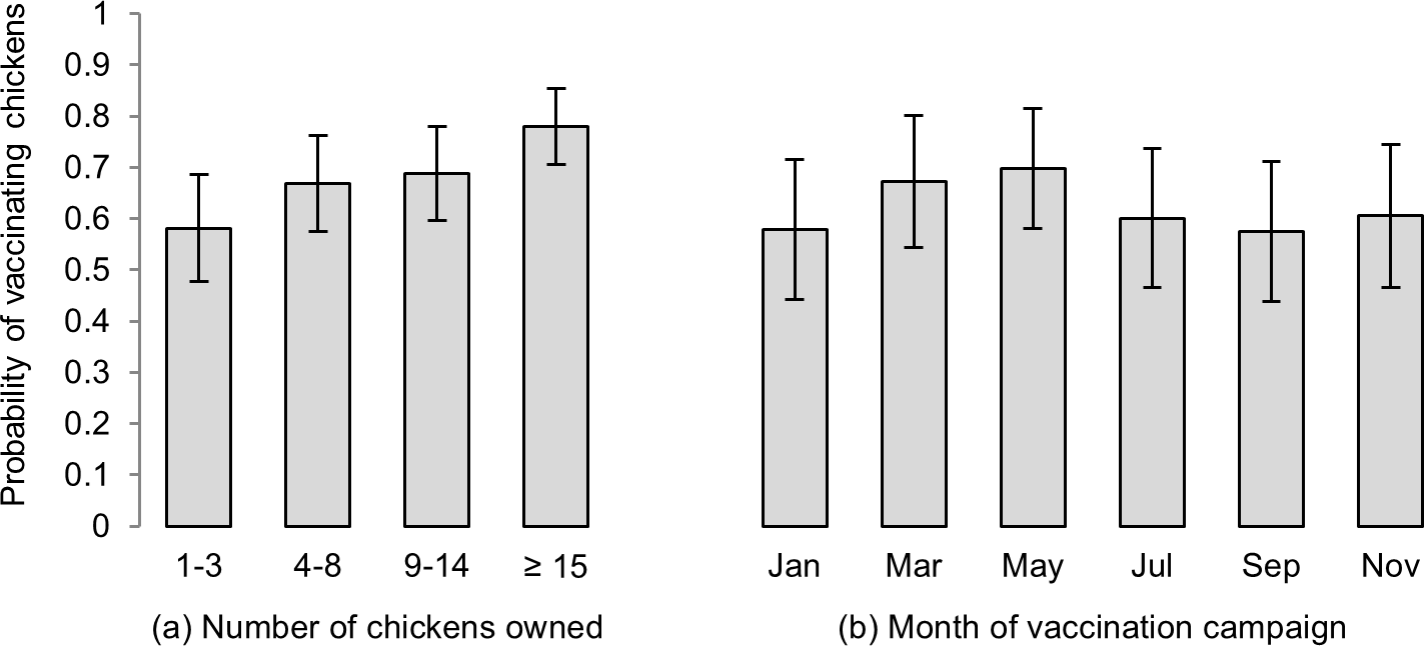
Probability of enrolled households participating in Newcastle disease vaccination campaigns, according to (a) quartiles of chicken flock size (above two months of age) at the time of vaccination (*p* < 0.001), and (b) the month of vaccination campaigns (*p* = 0.044).

Secondly, a binomial generalised linear mixed model was used to evaluate associations between the number of chickens owned by an enrolled household during the month of vaccination and the uptake of vaccination by that household. Allowing for variation between campaigns and geographic clustering within the dataset, a strong association was identified between larger flock size and vaccination uptake (*p* < 0.001). This was evident both considering chicken numbers as a continuous predictor variable, and evaluating quartiles of chicken flock size (Fig 7A). The latter approach, which demonstrates the majority of chicken flocks to be of a modest size, predicts a household with 15 or more chickens to have 2.6 times greater odds of vaccinating than a household with less than four chickens (0.792 probability of vaccinating vs. 0.598).

Within the same model, the probability of participating in a vaccination campaign was also shown to be significantly associated with the month of year (*p* = 0.044; Fig 5B). Although wide standard errors were associated with the model-based means based on this limited period of data collection, households were suggested to be most likely to vaccinate in the months of March and May, in Sanza and Majiri Wards, respectively.

**Fig 8:**
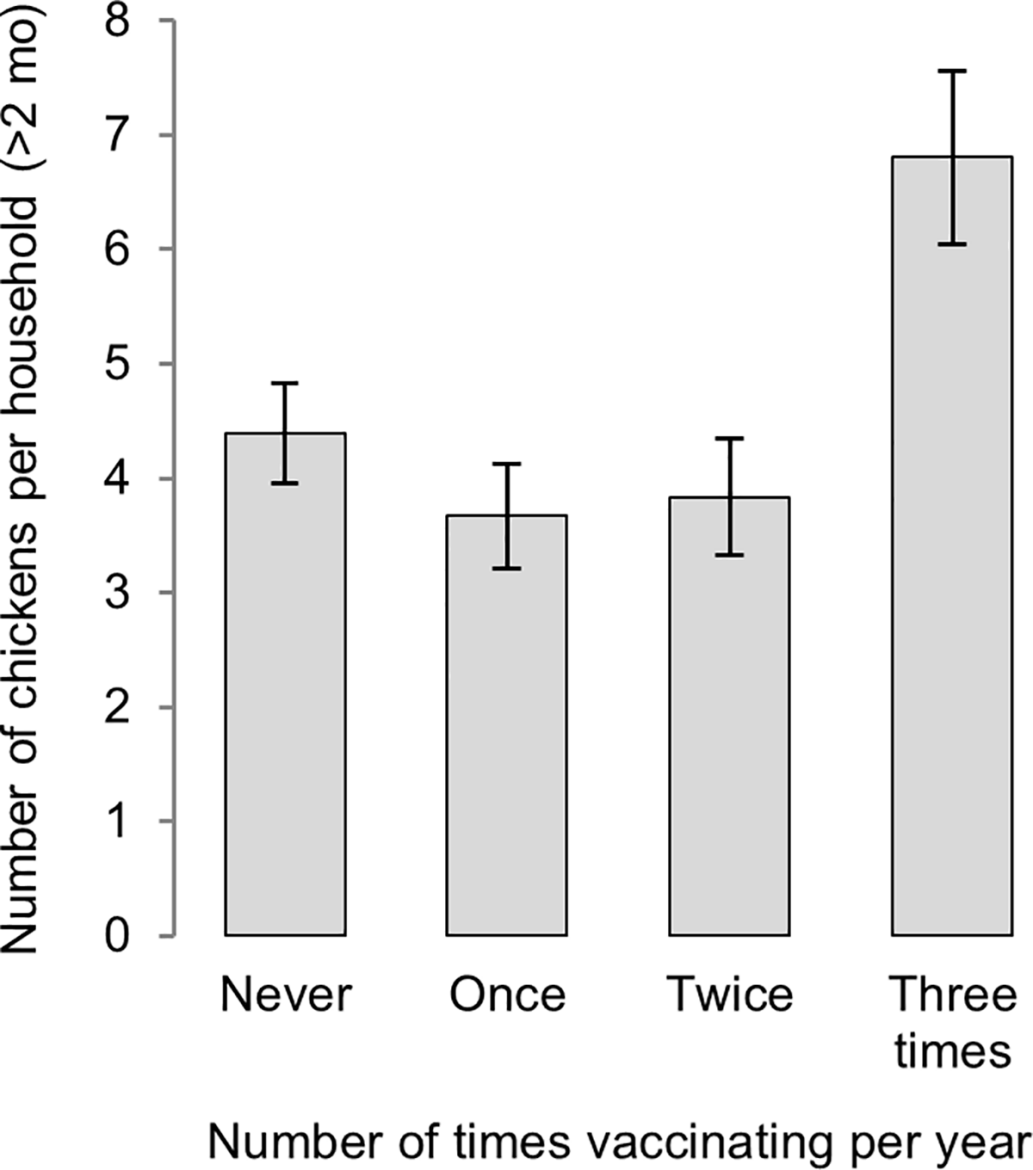
Model-based predictions for mean number of chickens (above two months of age) per household according to the number of campaigns in which chickens were vaccinated in the previous twelve months (*p* < 0.001), based on multivariable analysis of enrolled households (controlling for asset score).

A final component of the analysis explored the influence of participating in multiple vaccination campaigns on chicken flock size. Using two years of data from each of the two wards, the number of campaigns in which a household’s chickens were vaccinated over a twelve-month period (0–3) was evaluated as a predictor of the number of chickens owned at the end of this period. In a multivariable generalised linear mixed model, controlling for the influence of HDAI (*p* < 0.001), frequency of vaccination was significantly associated with chicken numbers (*p* < 0.001; Fig 8). Model-based predictions indicated households vaccinating in all three campaigns in a given twelve-month period to have almost twice as many chickens at the end of that period, as households vaccinating once, twice or not at all, for which predicted mean flock size did not differ significantly.

## Discussion

This study was devised with the aim of evaluating outcomes of a fee-for-service ND vaccination program on (i) vaccination uptake across the project area, and (ii) on levels of chicken ownership and flock sizes amongst enrolled households. This was considered in the context of a potential pathway to influence food security and the nutritional adequacy of diets for women and children within those households. Using a nationally-produced thermotolerant vaccine of known efficacy [29] and a “community vaccinator” model applied successfully elsewhere in the region [30], the emphasis of this work was on documenting the results of a cost-recovery chicken vaccination program on poultry flocks in rural communities of Tanzania.

The delayed project inception which led to the commencement of vaccination activities on the cusp of a high-risk period for ND outbreaks was a significant challenge. For chickens already harbouring the virus or suffering immunosuppression (due to high parasite burdens, poor diets or intercurrent disease) to fail to respond effectively to the vaccine and subsequently die would have severe implications for ongoing vaccination efforts in this ward. The decision to postpone payment in this first campaign was intended to minimise the potential for disease transmission via the handling of money, at a time when ND virus might be present within the community. It did, however, set a precedent of payment being delayed or being contingent on the ongoing good health of vaccinated birds, which has been difficult to overcome in some villages.

In the May 2014 campaign in Chicheho Village, of households not enrolled in the longitudinal study, the number recorded as having been vaccinated fractionally *exceeded* the total number of households in the village identified in the census (286 vaccinated, 285 households in total). Although levels of chicken ownership across the non-enrolled segment of the village is not known, only around half of the enrolled subset in Chicheho village (54.7%) kept chickens at this time. The deferred payment system and the commencement of activities in an area rarely visited by research or development programs in the past may have contributed to an elevated vaccination uptake, however it is unlikely that all non-enrolled households in the village might have owned chickens, let alone taken up the vaccination service in this first campaign.

Aside from the possibility of recording errors by newly-trained vaccinators, it is theorised that the apparently high vaccination rates in this village may reflect vaccination of households from outside the intervention area. Although distances between villages are not inconsequential, the potential that vaccinators may have travelled by bicycle or motorcycle to pursue income-earning opportunities further afield cannot be ruled out. The percentage of households keeping chickens and the mean flock size in the neighbouring village of Sanza are noted to have remained relatively stable during the high-risk period for ND outbreaks in this first year, in contrast to the fall in both ownership and chicken numbers in the other delayed intervention village of Ntope.

Signficantly higher levels of ND vaccination were observed amongst households enrolled in the longitudinal study, compared to others within the community (*p* = 0.009). This might be explained by either a higher supply or demand for the vaccination service amongst enrolled households. In terms of demand, Community Assistants’ twice-monthly documentation of the number of chickens owned, and a series of questions about chicken ownership and the consumption of chicken meat and eggs during six-monthly questionnaires, are likely to have generated greater interest in poultry-keeping amongst this subset–and potentially a greater likelihood of investing in vaccination. The targeted inclusion of enrolled households in extension activities and the provision of small gifts as tokens of appreciation for their involvement in the study are recognised as likely to have contributed to the increased uptake of vaccination, relative to non-enrolled households of equivalent economic capacity and language group. Additionally, on the supply front, close interactions between Community Assistants and chicken-vaccinators may have resulted in an increase in the vaccination service being offered to enrolled households.

Deeper exploration of factors influencing vaccination uptake and economic analyses of this ND control program (including the influence of an increase in the cost of vaccination in Majiri Ward in 2016) are beyond the scope of this article. Rather, in this paper, we seek to evaluate associations between vaccination at a household level and chicken flock size. When reviewing summaries of chicken ownership and mean flock size across enrolled households over a two-year period in the absence of information on the sale and consumption of chickens, outcomes attributable to the introduction of the ND vaccine are difficult to detect. This is unsurprising, in the early phase of an animal health program which requires households to pay for a novel technology and when a reduction in chicken mortality may be countered by their more frequent sale to meet household needs in times of climate variability, crop failure and a scarcity of staple foods. Rainfall in the first year of data collection was particularly poor, with a total rainfall of 447 mm (30 rain days) in Sanza Ward and 275 mm (21 rain days) in Majiri Ward, falling short of the long-term mean annual rainfall of 624 mm (49 rain days) for Manyoni District [19]. While not documented within this study, increased sale of chickens to meet household needs is likely to have occurred.

Despite limitations in the ability to capture a household’s socioeconomic status effectively using an index which has not been validated in the study setting, significant associations between increasing asset score and increasing chicken flock size were found. Chicken flocks were also shown to be significantly larger for households identifying as belonging to the Sukuma language group, compared to others within the study population. Controlling for variation due to socioeconomic status and language group, households vaccinating their chickens in a given campaign were identified to have larger chicken flocks in the period following vaccination. This finding is not only contingent on vaccine efficacy and its appropriate storage, handling and administration, but also on the fact that households within the study have an interest in increasing their chicken flock size–and that reduced mortality due to ND will not immediately be met by increased sale and consumption of chickens.

Determining causality is difficult through such analyses, and the possibility that households with larger chicken flocks have greater interest and financial capacity to invest in ND vaccination must also be considered. This additional hypothesis, that chicken flock size is a determinant of vaccination uptake, was also tested. The finding that households with larger flocks during the month of the vaccination campaign were more likely to have their chickens vaccinated may reflect the fact that community vaccinators preferentially offer their service to households with larger flocks. If payment is received for each bird vaccinated and distances between households substantial, there is an incentive for vaccinators to focus their efforts on households with a greater number of chickens. Previous findings from Malawi, Mozambique and Tanzania have highlighted the adequate compensation of vaccinators as being fundamental to the success of ND control programs [31].

Alternatively, there may be an increased demand for vaccination amongst households with more chickens, for whom poultry-keeping may be more likely to be seen as a viable livelihood strategy and who may be more willing or able to pay for the service. Suggested seasonal patterns in the probability of vaccinating are consistent with both the availability of income from crop sales and the reported patterns of ND outbreaks in this area. Higher levels of vaccination in the months of March and May reflect a time of greater income availability following the harvest of crops, and heightened awareness of the risk of ND outbreaks in the following months. In November and January, the lower perception of disease risk and scarce disposable income may explain the lower likelihood of households choosing to vaccinate their flocks.

The finding of significantly higher chicken flock size (*p* < 0.001) amongst households participating consistently in vaccination campaigns, compared to those vaccinating intermittently or not at all, reinforces the merit of the four-monthly vaccination protocol advocated for using the I-2 ND vaccine in village settings [21]. Although seasonal patterns exist for high-mortality ND outbreaks, benefits of regular vaccination include maintaining ongoing and adequate immunity to reduce the incidence of disease at other times of year, and contributing to herd immunity to protect chicks which are hatched between campaigns [20, 29].

## Conclusions

Animal health programs–and development programs more broadly–that involve the voluntary investment of funds by local households offer a sustainable pathway to food and nutrition security, which avoids a reliance on ongoing support from governments or donor agencies. Village chickens, ubiquitous throughout rural communities of sub-Saharan Africa, are a worthy target of efforts to alleviate poverty and enhance diet quality. Within this study, the finding of greater flock size following Newcastle disease vaccination provides assurance of the vaccine being handled and administered effectively by local vaccinators. Increased thermotolerance of the I-2 ND vaccine removes the need for a continuous cold chain, but requirements for appropriate storage, transport and ensuring opened vaccine vials are used within an appropriate time period remain important.

Equally central to a successful ND control program is the awareness-raising and educational role of the vaccinator within their community. Tanzanian poultry-keepers are familiar with clinical signs and patterns of disease amongst their birds, and, although formal diagnosis is rare, illness and mortality compatible with Newcastle disease (*Mdondo* in Swahili) is a known entity. Despite this, when financial reserves are scarce, climate patterns unpredictable, available environmental feed resources limited and the threat of predation and theft common, poultry-keepers may be initially reluctant to invest in ND vaccination programs. This study’s finding of increased vaccination uptake by households to whom questions about chicken ownership, management and consumption were posed on a regular basis supports the idea of awareness generating demand for vaccination.

As per the age-old “chicken or the egg” conundrum, linkages between vaccination and poultry flock size are confirmed to be bi-directional: vaccination leads to increased flock size, and larger flocks are more likely to be vaccinated. The real dilemma is how to encourage vaccination amongst households with fewer chickens and to support chicken-keepers to vaccinate regularly. One targeted program in Mozambique has involved the distribution of both chickens and ND vaccination vouchers to households affected by HIV/AIDS, allowing community vaccinators to collect payment for their service from two local non-governmental organisations during the first year of a newly introduced ND vaccination program [32]. Opportunities for a similar subsidised vaccination service for vulnerable households elsewhere in Africa, perhaps integrated with existing or future social welfare programs, warrants further exploration. Qualitative approaches to explore household decision-making around chicken health and management, allocation of income, and motivations, challenges and priorities of community vaccinators will be central to the identification and addressing of barriers to broader vaccination coverage.

Findings which link chicken flock size with households’ socioeconomic status affirm the value of integrated approaches to poverty alleviation in efforts to enhance food and nutrition security. There is scope for greater economic and nutritional contributions of village chickens, but this may be accelerated when other strategies to increase the household income are in place. In the context of the broader interdisciplinary nutrition-sensitive program of which this study forms a part, the synergistic outcomes of improved crop production and poultry health are proposed to contribute to increased household income, less vulnerability to extreme weather events, greater willingness and capacity to invest in crop and poultry production and, ultimately, improved human nutrition outcomes.

## Supporting information

S1 Table Output from linear mixed model for mean chicken flock size in the period between one Newcastle disease vaccination campaign and the next. (DOCX)

S2 Table Output from generalised linear mixed model (binomial) for participation in a given Newcastle disease vaccination campaign. (DOCX)

S3 Table Output from generalised linear mixed model (Poisson distribution) for chicken flock size at the end of a twelve month period. (DOCX)

S1 Dataset Data used for analyses in this manuscript. (XLSX)
