# Supplementary material for: The chicken or the egg? Exploring bi-directional associations between Newcastle disease vaccination and village chicken flock size in rural Tanzania
Source: PLoS One. 2017 Nov 16;12(11):e0188230. doi: 10.1371/journal.pone.0188230 (PMC5690622; doi:10.1371/journal.pone.0188230)
Supplement: S1 Table — (DOCX) [file pone.0188230.s001.docx]

**S1 Table: Output from linear mixed model for mean chicken flock size in the period between one Newcastle disease vaccination campaign and the next.**

| *Outcome: Mean chicken flock size between ND vaccination campaigns* | | | |
| --- | --- | --- | --- |
| Fixed effect | | Regression coeff. | SE |
| Constant | | 1.151 | 0.132 |
| log*_e_*(Household domestic asset index+0.5) | | 0.045 | 0.017 |
|  | | Predicted mean | SE |
| Language group | Sukuma | 2.378 | 0.147 |
|  | Other | 1.854 | 0.090 |
| Vaccinated | Yes | 2.226 | 0.105 |
|  | No | 2.007 | 0.106 |
| Campaign | May 2014 | 1.744 | 0.129 |
|  | Sep 2014 | 2.001 | 0.133 |
|  | Jan 2015 | 2.184 | 0.116 |
|  | Mar 2015 | 2.244 | 0.208 |
|  | May 2015 | 2.256 | 0.115 |
|  | Jul 2015 | 2.266 | 0.127 |
|  | Sep 2015 | 2.07 | 0.130 |
|  | Nov 2015 | 1.946 | 0.125 |
|  | Jan 2016 | 2.105 | 0.122 |
|  | Mar 2016 | 1.949 | 0.128 |
|  | May 2016 | 2.203 | 0.121 |
|  | Jul 2016 | 2.371 | 0.125 |
|  | Sep 2016 | 2.171 | 0.120 |
| Vaccinated.Campaign | Yes, May 2014 | 2.075 | 0.161 |
|  | Yes, Sep 2014 | 1.943 | 0.173 |
|  | Yes, Jan 2015 | 2.170 | 0.146 |
|  | Yes, Mar 2015 | 2.532 | 0.151 |
|  | Yes, May 2015 | 2.389 | 0.139 |
|  | Yes, Jul 2015 | 2.439 | 0.132 |
|  | Yes, Sep 2015 | 2.139 | 0.175 |
|  | Yes, Nov 2015 | 2.069 | 0.134 |
|  | Yes, Jan 2016 | 2.174 | 0.141 |
|  | Yes, Mar 2016 | 2.071 | 0.138 |
|  | Yes, May 2016 | 2.279 | 0.132 |
|  | Yes, Jul 2016 | 2.499 | 0.137 |
|  | Yes, Sep 2016 | 2.152 | 0.142 |
|  | No, May 2014 | 1.412 | 0.140 |
|  | No, Sep 2014 | 2.060 | 0.142 |
|  | No, Jan 2015 | 2.198 | 0.116 |
|  | No, Mar 2015 | 1.957 | 0.358 |
|  | No, May 2015 | 2.122 | 0.120 |
|  | No, Jul 2015 | 2.093 | 0.161 |
|  | No, Sep 2015 | 2.001 | 0.127 |
|  | No, Nov 2015 | 1.822 | 0.152 |
|  | No, Jan 2016 | 2.036 | 0.137 |
|  | No, Mar 2016 | 1.828 | 0.159 |
|  | No, May 2016 | 2.127 | 0.143 |
|  | No, Jul 2016 | 2.243 | 0.150 |
|  | No, Sep 2016 | 2.190 | 0.132 |
| Random effect | | Variance | SE |
| Ward | | 0 | - |
| Ward.Village | | 0.040 | 0.035 |
| Ward.Village.Subvillage | | 0.022 | 0.024 |
| Ward.Village.Subvillage.ID | | 0.485 | 0.049 |
