# Supplementary material for: The chicken or the egg? Exploring bi-directional associations between Newcastle disease vaccination and village chicken flock size in rural Tanzania
Source: PLoS One. 2017 Nov 16;12(11):e0188230. doi: 10.1371/journal.pone.0188230 (PMC5690622; doi:10.1371/journal.pone.0188230)
Supplement: S2 Table — (DOCX) [file pone.0188230.s002.docx]

**S2 Table: Output from generalised linear mixed model (binomial) for participation in a given Newcastle disease vaccination campaign.**

| *Outcome: Participation in a given ND vaccination campaign* | | | |
| --- | --- | --- | --- |
| Fixed effect | | Regression coeff. | SE |
| Constant | | 0.317 | 0.559 |
| log*_e_*(No. chickens in vaccination month+0.5) | | 0.346 | 0.082 |
|  | | Predicted probability | SE |
| Vaccination month | Jan | 0.317 | 0.559 |
|  | Mar | 0.721 | 0.583 |
|  | May | 0.833 | 0.554 |
|  | Jul | 0.410 | 0.565 |
|  | Sep | 0.300 | 0.560 |
|  | Nov | 0.428 | 0.585 |
| Random effect | | Variance | SE |
| Ward | | 0.254 | 0.860 |
| Ward.Village | | 1.026 | 0.765 |
| Ward.Village.Subvillage | | 0.804 | 0.327 |
| Ward.Village.Subvillage.ID | | 0.942 | 0.187 |
