# Supplementary material for: The chicken or the egg? Exploring bi-directional associations between Newcastle disease vaccination and village chicken flock size in rural Tanzania
Source: PLoS One. 2017 Nov 16;12(11):e0188230. doi: 10.1371/journal.pone.0188230 (PMC5690622; doi:10.1371/journal.pone.0188230)
Supplement: S3 Table — (DOCX) [file pone.0188230.s003.docx]

**S3 Table: Output from generalised linear mixed model (Poisson distribution) for chicken flock size at the end of a twelve month period.**

| *Outcome: Number of chickens at end of twelve month period* | | | |
| --- | --- | --- | --- |
| Fixed effect | | Regression coeff. | SE |
| Constant | | 1.480 | 0.0989 |
| log*_e_*(Household domestic asset index+0.5) | | 0.116 | 0.026 |
|  | | Predicted mean | SE |
| Times vaccinated | Never | 1.480 | 0.099 |
|  | Once | 1.300 | 0.125 |
|  | Twice | 1.344 | 0.133 |
|  | Three times | 1.918 | 0.111 |
| Random effect | | Variance | SE |
| Ward | | 0.000 | - |
| Ward.Village | | 0.040 | 0.034 |
| Ward.Village.Subvillage | | 0.004 | 0.023 |
| Ward.Village.Subvillage.ID | | 0.330 | 0.063 |
